# Association of nucleotide excision repair pathway gene polymorphisms with gastric cancer and atrophic gastritis risks

**DOI:** 10.18632/oncotarget.6853

**Published:** 2016-01-09

**Authors:** Jingwei Liu, Liping Sun, Qian Xu, Huakang Tu, Caiyun He, Chengzhong Xing, Yuan Yuan

**Affiliations:** ^1^ Tumor Etiology and Screening Department of Cancer Institute and General Surgery, the First Affiliated Hospital of China Medical University, Shenyang 110001, China; ^2^ Key Laboratory of Cancer Etiology and Prevention, China Medical University, Liaoning Provincial Education Department, Shenyang 110001, China

**Keywords:** nucleotide excision repair, gastric cancer, polymorphism

## Abstract

Polymorphisms of NER genes could change NER ability, thereby altering individual susceptibility to GC. We systematically analyzed 39 SNPs of 8 key genes of NER pathway in 2686 subjects including 898 gastric cancer (GC), 851 atrophic gastritis (AG) and 937 controls (CON) in northern Chinese. SNP genotyping were performed using Sequenom MassARRAY platform. The results demonstrated that *DDB2* rs830083 GG genotype was significantly associated with increased GC risk compared with wildtype CC (OR=2.32, P = 6.62 × 10^−9^); *XPC* rs2607775 CG genotype conferred a 1.73 increased odds of GC risk than non-cancer subjects compared with wild-type CC (OR=1.73, P= 3.04 × 10^−4^). The combined detection of these two polymorphisms demonstrated even higher GC risk (OR=3.05). Haplotype analysis suggested that *DDB2* rs2029298-rs326222-rs3781619-rs830083 GTAG haplotype was significantly associated with disease risk in each step of CON→AG→GC development (AG vs. CON: OR=2.88, P= 7.51 × 10^−7^; GC vs. AG: OR=2.90, P=5.68 × 10^−15^; GC vs. CON: OR=8.42, P=2.22 × 10^−15^); *DDB2* GTAC haplotype was associated with reduced risk of GC compared with CON (OR=0.63, P= 8.31 × 10^−12^). *XPC* rs1870134-rs2228000- rs2228001-rs2470352-rs2607775 GCAAG haplotype conferred increased risk of GC compared with AG (OR=1.88, P= 6.98 × 10^−4^). *XPA* rs2808668 and drinking, *DDB2* rs326222, rs3781619, rs830083 and smoking demonstrated significant interactions in AG; *XPC* rs2607775 had significant interaction with smoking in GC. In conclusion, NER pathway polymorphisms especially in “damage incision” step were significantly associated with GC risk and had interactions with environment factors. The detection of NER pathway polymorphisms such as *DDB2* and *XPC* might be applied in the prediction of GC risk and personalized prevention in the future.

**NOVELTY & IMPACT STATEMENTS:**

NER pathway polymorphisms especially in “damage incision” step were significantly associated with GC risk and had interactions with environment factors, which might be applied in the prediction of GC risk and personalized prevention in the future.

## INTRODUCTION

Gastric cancer (GC) is one of the most common cancers and is the second leading cause of cancer-related death worldwide [1]. Disease development progresses stepwise from a normal stomach through inflammation and precancerous conditions to cancer, as described by Correa's cascade [2]. Although environmental factors such as *Helicobacter pylori* infection are known risk factors for GC, genetic influences and interactions with environmental factors also play an essential role in its initiation [3]. Therefore, the screening and identification of genetic factors that are associated with risks of GC and its precancerous diseases would reveal the etiology and pathogenesis.

As the most common form of genetic variation, single nucleotide polymorphisms (SNP) have been widely investigated in relation to the risk of cancers. Genome-wide association studies (GWAS) have identified several SNPs that are significantly associated with high GC risk including: *MUC1* rs2070803 G/A and *PSCA* rs2976392 A/G, associated with an increased risk of diffuse-type GC in a Japanese population (odds ratio (OR)=1.63, *P* =1.2 × 10^−6^; OR=1.62, *P* =1.1 × 10^−9^) [4]; *PLCE1* rs2274223 A/G, associated with a high GC risk in a Chinese population (OR=1.31, *P* =8.4 × 10^−9^) [5]; and *PRKAA1* rs13361707 T/C, which was associated with an increased risk of non-cardia GC (OR=1.41, *P* =7.6 × 10^−29^) [6]. A number of candidate gene association studies have also identified SNPs in genes encoding pepsinogen C and glutathione S-transferase pi 1, which appear to significantly alter individual susceptibility to GC [7, 8]. Although these studies have found several SNPs related to GC risk, most focused on scattered SNPs rather than integral gene-gene pathways or gene-environment interactions. Thus, the screening of additional key SNPs is still required to elucidate their role in different stages of gastric carcinogenesis.

Nucleotide excision repair (NER) is a versatile system that monitors and repairs DNA damage, including ultraviolet (UV)-induced cyclobutane pyrimidine dimers, DNA crosslinks, and bulky adducts [9]. NER stages include damage recognition, damage demarcation and unwinding, damage incision, and new strand ligation [10], all of which require corresponding functional proteins. Cellular DNA is constantly at risk from damage by endogenous and exogenous stimuli, and NER defects are likely to increase genome instability [11]. Polymorphisms of NER genes might change the NER ability by influencing the expression and function of key proteins, thereby altering individual susceptibility to GC and giving rise to gastric carcinogenesis[12, 13].

Polymorphisms of several key NER genes have previously been reported to alter the GC risk, including xeroderma pigmentosum, complementation group A (*XPA*) and xeroderma pigmentosum, complementation group C (*XPC*) in the damage recognition step, excision repair cross-complementation group 2 (*ERCC2*) in the damage unwinding step, and *ERCC1*, *ERCC4*, and *ERCC5* in the damage incision step [13]. However, most of these studies investigated only a few SNPs of a single gene. For instance, Chen et al. reported three *ERCC2* SNPs [14], while He et al. studied three *ERCC5* SNPs [12]. No study has yet analyzed the role of SNPs from the entire NER pathway in gastric carcinogenesis. In the present study, therefore, we systematically analyzed 39 SNPs of eight key NER genes (*ERCC1*, *ERCC2*, *ERCC3*, *ERCC4*, *ERCC5*, *XPA*, *XPC*, and the damage-specific DNA binding protein 2 gene *DDB2*) in a total of 2686 northern Chinese subjects including 898 with GC, 851 with atrophic gastritis (AG), and 937 controls. The roles of these SNPs at different stages of gastric carcinogenesis as well as gene-gene and gene-environment interactions were investigated to determine whether they could be used to predict GC risk.

## RESULTS

### Characteristics of subjects

A total of 2686 subjects were selected for inclusion in the study, and their characteristics are shown in Supplementary Table S2. GC and AG groups had a significantly higher proportion of males (71.2 and 56.2%, respectively) compared with the control (CON) group (54.4%; *P* < 0.001). *H. pylori* infection rates (50.6 and 50.9%, respectively) were also significantly higher in GC and AG groups than the CON group (29.2%; *P* < 0.001). Based on Lauren's histological classification of GC, 269 cases were intestinal-type (37.3%) and 453 cases were diffuse-type (62.7%).

### NER pathway gene SNPs and disease risk

Our study focused on 39 SNPs in eight NER pathway genes (*ERCC1*, *ERCC2*, *ERCC3*, *ERCC4*, *ERCC5*, *XPA*, *XPC*, and *DDB2*). Six SNPs were not in accordance with Hardy-Weinberg equilibrium (HWE) so were not entered into the subsequent association study. Primary details of SNPs and allele frequencies are listed in Supplementary Table S1 and the associations of genotypes with disease risk by different genetic models are summarized in Supplementary Table S3. Subgroup analysis results for *H. pylori* infection positive/negative and intestinal/diffuse-type GC are shown in Supplementary Tables S4 and Table S5, respectively.

*XPA* SNPs rs10817938, rs2808668 and *DDB2* SNP rs830083 were found to be significantly associated with the risk of AG, while *XPC* SNP rs2607775 and *DDB2* SNPs rs2029298, rs326222, rs3781619, and rs830083 were significantly associated with GC risk (Table 1). After Bonferroni correction for multiple comparisons, *DDB2* rs830083 and *XPC* rs2607775 remained significantly associated with increased GC risk: the *DDB2* rs830083 GG genotype was significantly associated with an increased risk of GC compared with the wild-type CC genotype (OR=2.32, 95% confidence interval (CI)=1.75-3.08, *P* =6.62 × 10^−9^), and the *XPC* rs2607775 CG genotype conferred a 1.73-fold increased GC risk compared with the wild-type CC genotype (OR=1.73, 95%CI=1.28-2.32, *P* =3.04 × 10^−4^).

**Table 1:** NER SNPs that demonstrate significant association with disease risk

	Gastric mucosa status			Compared	CON→AG		AG→GC		CON→GC		Non-cancer→GC	
SNP	GC(%)	AG(%)	CON(%)	Genotype	OR(95%CI)	P	OR(95%CI)	P	OR(95%CI)	P	OR(95%CI)	P
XPA rs10817938	547(61.4)	488(57.7)	595(63.8)	TT	ref.		ref.		ref.		ref.	
	307(34.5)	318(37.6)	299(32.1)	CT	**1.34(1.09-1.66)**	**0.006**	0.86(0.70-1.06)	0.153	1.10(0.89-1.36)	0.392	0.95(0.80-1.14)	0.585
	37(4.2)	40(4.7)	38(4.1)	CC	1.43(0.88-2.31)	0.150	0.86(0.54-1.39)	0.545	1.29(0.78-2.12)	0.327	1.06(0.70-1.60)	0.794
				Dominant	**1.35(1.10-1.65)**	**0.004**	0.86(0.71-1.05)	0.139	1.11(0.91-1.37)	0.302	0.96(0.81-1.14)	0.658
				Recessive	1.28(0.79-2.07)	0.308	0.91(0.57-1.46)	0.703	1.23(0.75-2.01)	0.418	1.07(0.71-1.62)	0.746
				Additive	**1.29(1.08-1.53)**	**0.004**	0.89(0.75-1.05)	0.154	1.11(0.93-1.32)	0.235	0.98(0.85-1.13)	0.787
XPA rs2808668	216(24.3)	243(28.8)	224(24.1)	TT	ref.		ref.		ref.		ref.	
	435(48.9)	401(47.6)	482(51.9)	CT	**0.74(0.58-0.94)**	**0.015**	1.26(1.00-1.59)	0.057	0.90(0.71-1.16)	0.421	1.08(0.88-1.33)	0.462
	239(26.9)	199(23.6)	223(24.0)	CC	0.81(0.61-1.06)	0.127	**1.35(1.03-1.76)**	**0.029**	1.04(0.79-1.38)	0.773	1.23(0.98-1.56)	0.080
				Dominant	**0.76(0.61-0.96)**	**0.018**	**1.29(1.03-1.60)**	**0.024**	0.95(0.75-1.20)	0.658	1.13(0.93-1.37)	0.211
				Recessive	0.96(0.77-1.21)	0.751	1.17(0.94-1.47)	0.161	1.12(0.90-1.41)	0.314	1.18(0.97-1.43)	0.092
				Additive	0.89(0.77-1.02)	0.091	**1.17(1.02-1.34)**	**0.025**	1.02(0.89-1.18)	0.765	1.11(0.99-1.25)	0.078
XPC rs2607775	802(89.5)	795(94.0)	864(92.4)	CC	ref.		ref.		ref.		ref.	
	91(10.2)	51(6.0)	70(7.5)	CG	0.76(0.51-1.12)	0.166	**1.88(1.30-2.72)**	**7.35 × 10^−4^**	**1.52(1.07-2.15)**	**0.019**	**1.73(1.28-2.32)**	**3.04 ×10^−4^**
	3(0.3)	0(0.0)	1(0.1)	GG	/	/	/	/	1.37(0.14-13.55)	0.786	3.00(0.31-29.38)	0.346
				Dominant	0.75(0.50-1.11)	0.145	**1.92(1.33-2.77)**	**4.56 × 10^−4^**	**1.51(1.07-2.14)**	**0.019**	**1.74(1.30-2.34)**	**2.09 × 10^−4^**
				Recessive	/	/	/	/	1.32(0.13-12.97)	0.814	2.86(0.29-27.99)	0.367
				Additive	0.76(0.51-1.12)	0.165	**1.92(1.34-2.76)**	**3.90 × 10^−4^**	**1.52(1.08-2.13)**	**0.016**	**1.74(1.30-2.32)**	**1.62 × 10^−4^**
DDB2 rs2029298	389(43.7)	399(47.2)	451(48.2)	GG	ref.		ref.		ref.		ref.	
	421(47.3)	350(41.4)	389(41.6)	AG	1.04(0.85-1.28)	0.705	1.21(0.99-1.49)	0.069	1.19(0.97-1.47)	0.101	**1.21(1.02-1.45)**	**0.031**
	81(9.1)	96(11.4)	95(10.2)	AA	1.10(0.79-1.54)	0.564	0.85(0.61-1.18)	0.324	0.98(0.69-1.39)	0.908	0.90(0.67-1.20)	0.459
				Dominant	1.05(0.87-1.28)	0.609	1.13(0.93-1.37)	0.213	1.15(0.95-1.40)	0.163	1.15(0.97-1.36)	0.108
				Recessive	1.09(0.79-1.50)	0.603	0.77(0.56-1.06)	0.106	0.90(0.64-1.26)	0.527	0.81(0.61-1.07)	0.139
				Additive	1.04(0.90-1.21)	0.602	1.02(0.88-1.18)	0.815	1.05(0.91-1.23)	0.500	1.03(0.91-1.17)	0.611
DDB2 rs326222	457(50.9)	436(51.8)	480(51.4)	TT	ref.		ref.		ref.		ref.	
	392(43.7)	330(39.2)	393(42.1)	CT	0.99(0.80-1.22)	0.903	1.11(0.90-1.36)	0.324	1.02(0.83-1.25)	0.879	1.07(0.90-1.27)	0.476
	48(5.4)	75(8.9)	61(6.5)	CC	1.42(0.97-2.08)	0.073	**0.64(0.43-0.94)**	**0.025**	0.82(0.53-1.26)	0.356	0.71(0.50-1.02)	0.065
				Dominant	1.05(0.86-1.27)	0.665	1.02(0.84-1.24)	0.818	0.99(0.81-1.21)	0.921	1.01(0.86-1.19)	0.899
				Recessive	1.43(0.99-2.08)	0.058	**0.61(0.41-0.89)**	**0.011**	0.81(0.53-1.23)	0.317	**0.69(0.49-0.98)**	**0.039**
				Additive	1.09(0.93-1.27)	0.300	0.94(0.80-1.09)	0.402	0.96(0.81-1.13)	0.588	0.95(0.83-1.09)	0.446
DDB2 rs3781619	347(38.9)	342(40.5)	391(41.9)	AA	ref.		ref.		ref.		ref.	
	451(50.5)	380(45.0)	419(44.9)	AG	1.10(0.89-1.36)	0.402	1.15(0.94-1.42)	0.179	1.18(0.95-1.45)	0.136	1.18(0.99-1.41)	0.072
	95(10.6)	123(14.6)	124(13.3)	GG	1.12(0.83-1.53)	0.452	0.76(0.55-1.04)	0.081	0.88(0.64-1.22)	0.440	0.81(0.62-1.07)	0.135
				Dominant	1.10(0.90-1.34)	0.355	1.06(0.87-1.29)	0.582	1.11(0.91-1.35)	0.321	1.09(0.92-1.29)	0.318
				Recessive	1.08(0.81-1.43)	0.615	**0.70(0.52-0.94)**	**0.017**	0.80(0.59-1.09)	0.162	**0.74(0.57-0.96)**	**0.022**
				Additive	1.06(0.92-1.23)	0.405	0.95(0.82-1.09)	0.459	1.00(0.86-1.16)	0.998	0.97(0.86-1.10)	0.683
DDB2 rs830083	258(29.0)	310(36.6)	374(40.0)	CC	ref.		ref.		ref.		ref.	
	410(46.1)	371(43.9)	423(45.3)	CG	1.12(0.90-1.39)	0.323	**1.28(1.02-1.60)**	**0.032**	**1.34(1.07-1.68)**	**0.011**	**1.33(1.09-1.61)**	**0.004**
	222(24.9)	165(19.5)	137(14.7)	GG	**1.48(1.11-1.97)**	**0.008**	**1.63(1.25-2.13)**	**3.29 × 10^−4^**	**2.32(1.75-3.08)**	**6.62 × 10^−9^**	**1.94(1.54-2.44)**	**2.18 × 10^−8^**
				Dominant	1.20(0.98-1.48)	0.073	**1.39(1.13-1.71)**	**0.002**	**1.58(1.28-1.95)**	**2.05 × 10^−5^**	**1.49(1.25-1.79)**	**1.19 × 10^−5^**
				Recessive	**1.40(1.08-1.82)**	**0.012**	**1.42(1.12-1.80)**	**0.003**	**1.96(1.52-2.53)**	**2.06 × 10^−7^**	**1.65(1.35-2.02)**	**1.33 × 10^−6^**
				Additive	**1.19(1.04-1.37)**	**0.013**	**1.28(1.12-1.46)**	**2.98 × 10^−4^**	**1.49(1.30-1.72)**	**2.13 × 10^−8^**	**1.38(1.23-1.55)**	**3.88 × 10^−8^**

The association between each SNP and risk of AG and GC was estimated by calculating ORs and their 95% CIs by multivariate logistic regression with adjustments for gender, age, and *H. pylori* infection status.

Abbreviations: GC, gastric cancer; AG, atrophic gastritis; CON, control.

**Figure 1:**
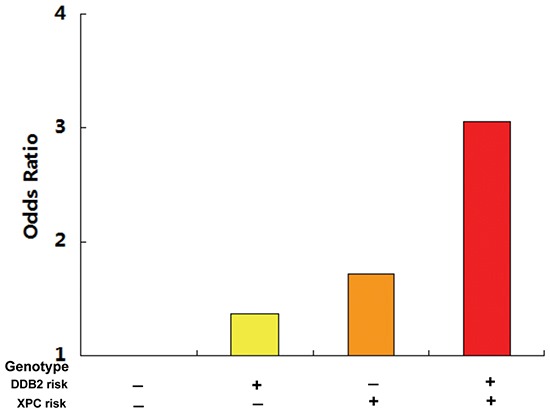
The combined detection of the DDB2 rs830083 and XPC rs2607775 risk genotypes demonstrated an even higher GC risk, with individuals carrying both risk genotypes having a 3.05-fold increased risk of developing GC

The combined analysis of the *DDB2* rs830083 and *XPC* rs2607775 risk genotypes demonstrated an even higher GC risk, with individuals carrying both risk genotypes having a 3.05-fold increased risk of developing GC (Figure 1).

### Stratified analysis for DDB2 rs830083 and XPC rs2607775

We next performed stratified analyses of *DDB2* rs830083 and *XPC* rs2607775 by gender, presence of *H. pylori* infection, Lauren's classification of GC, and smoking and alcohol consumption status of individuals. As shown in Table 2, the association of the *DDB2* rs830083 GG genotype with GC risk was more obvious in subgroups of males (OR=2.61, *P* =4.26 × 10^−7^), non-smokers (OR=2.39, *P* =3.93 × 10^−4^), and non-drinkers (OR=3.10, *P* =3.41 × 10^−6^) than the controls. Individuals both positive and negative for *H. pylori* infection, and with either intestinal-type or diffuse-type all demonstrated an increased risk of GC if they also possessed the GG genotype. For *XPC* rs2607775, the CG genotype was significantly associated with an increased risk of GC in males (OR=2.00, *P* =4.03 × 10^−4^) and *H. pylori*-negative (OR=1.98, *P* =8.93 × 10^−4^) subgroups.

**Table 2:** Subgroup analysis of *DDB2* rs830083 and *XPC* rs2607775 polymorphisms

	CON→AG			AG→GC			CON→GC			Non-cancer→GC		
Group	OR(95%CI)	P	P_B-D test_	OR(95%CI)	P	P_B-D test_	OR(95%CI)	P	P_B-D test_	OR(95%CI)	P	P_B-D test_
*DDB2* rs830083 GG vs. CC												
All	1.48(1.11-1.97)	0.008		**1.63(1.25-2.13)**	**3.29 × 10^−4^**		**2.32(1.75-3.08)**	**6.62 × 10^−9^**		**1.94(1.54-2.44)**	**2.18 × 10^−8^**	
Gender			0.960			0.605			0.646			0.589
Male	1.67(1.11-2.50)	0.013		**1.75(1.24-2.46)**	**1.44 × 10^−3^**		**2.61(1.80-3.78)**	**4.26 × 10^−7^**		**2.10(1.57-2.82)**	**6.66 × 10^−7^**	
Female	1.35(0.89-2.04)	0.157		1.45(0.95-2.23)	0.086		1.97(1.26-3.08)	0.003		1.68(1.15-2.47)	0.008	
Hp infection			0.673			0.190			0.418			0.432
Positive	1.37(0.88-2.11)	0.162		**1.91(1.34-2.72)**	**3.53 × 10^−4^**		**2.70(1.71-4.26)**	**1.89 × 10^−5^**		**2.14(1.54-2.97)**	**5.75 × 10^−6^**	
Negative	1.59(1.08-2.32)	0.018		1.32(0.88-1.98)	0.181		**2.09(1.45-3.02)**	**7.49 × 10^−5^**		**1.75(1.26-2.43)**	**8.01 × 10^−4^**	
Lauren's classification						0.914			0.915			0.907
Intestinal	1.48(1.11-1.97)	0.008		1.82(1.23-2.69)	0.003		**2.75(1.82-4.17)**	**1.79 × 10^−6^**		**2.18(1.51-3.14)**	**2.91 × 10^−5^**	
Diffuse	1.48(1.11-1.97)	0.008		**1.70(1.24-2.34)**	**1.02 × 10^−3^**		**2.40(1.71-3.36)**	**3.45 × 10^−7^**		**2.04(1.52-2.72)**	**1.6 × 10^−6^**	
Smoking			0.013			<0.001			0.271			0.011
Smoker	2.56(1.30-5.07)	0.007		0.91(0.48-1.73)	0.770		2.01(1.00-4.04)	0.050		1.30(0.75-2.28)	0.352	
Nonsmoker	0.67(0.42-1.07)	0.094		**3.17(1.96-5.13)**	**2.63 × 10^−6^**		**2.39(1.48-3.88)**	**3.93 × 10^−4^**		**2.79(1.84-4.23)**	**1.24 × 10^−6^**	
Drinking			0.861			0.094			0.111			0.061
Drinker	1.59(0.71-3.53)	0.258		1.27(0.60-2.68)	0.535		1.51(0.71-3.18)	0.282		1.41(0.75-2.65)	0.283	
Nondrinker	0.94(0.60-1.45)	0.762		**2.99(1.88-4.76)**	**4.10 × 10^−6^**		**3.10(1.92-5.00)**	**3.41 × 10^−6^**		**2.97(1.98-4.48)**	**1.77 × 10^−7^**	
*XPC* rs2607775 CG vs. CC												
All	0.76(0.51-1.12)	0.166		**1.88(1.30-2.72)**	**7.35 × 10^−4^**		1.52(1.07-2.15)	0.019		**1.73(1.28-2.32)**	**3.04 × 10^−4^**	
Gender			0.812			0.259			0.336			0.220
Male	0.79(0.44-1.43)	0.436		2.19(1.33-3.61)	0.002		1.84(1.15-2.92)	0.010		**2.00(1.36-2.94)**	**4.03 × 10^−4^**	
Female	0.74(0.43-1.25)	0.259		1.51(0.86-2.65)	0.150		1.14(0.66-1.98)	0.644		1.34(0.83-2.18)	0.236	
Hp infection			0.109			0.595			0.016			0.121
Hp(+)	0.58(0.33-1.00)	0.051		1.79(1.08-2.96)	0.025		0.99(0.58-1.70)	0.975		1.44(0.93-2.22)	0.105	
Hp(−)	1.01(0.59-1.73)	0.968		1.95(1.14-3.33)	0.015		2.01(1.28-3.16)	0.002		**1.98(1.32-2.97)**	**8.93 × 10^−4^**	
Lauren's classification						0.747			0.733			0.714
Intestinal	0.76(0.51-1.12)	0.166		2.07(1.24-3.48)	0.006		1.52(0.91-2.54)	0.108		1.85(1.17-2.92)	0.009	
Diffuse	0.76(0.51-1.12)	0.166		1.71(1.11-2.62)	0.015		1.33(0.87-2.02)	0.185		1.55(1.07-2.25)	0.021	
Smoking			0.876			0.099			0.041			0.027
Smoker	1.01(0.33-3.09)	0.981		2.91(1.09-7.75)	0.033		3.02(1.22-7.43)	0.016		2.92(1.38-6.17)	0.005	
Nonsmoker	0.71(0.40-1.27)	0.245		1.34(0.70-2.56)	0.383		0.91(0.48-1.71)	0.766		1.17(0.67-2.04)	0.573	
Drinking			0.420			0.247			0.665			0.397
Drinker	0.49(0.14-1.71)	0.263		3.23(0.99-10.56)	0.053		1.58(0.63-3.98)	0.336		1.98(0.87-4.52)	0.103	
Nondrinker	0.83(0.47-1.47)	0.529		1.51(0.81-2.82)	0.194		1.25(0.68-2.33)	0.473		1.46(0.85-2.49)	0.172	

The association between each SNP and risk of AG and GC was estimated by calculating ORs and their 95% CIs by multivariate logistic regression with adjustments for gender, age, and *H. pylori* infection status.

Abbreviations: GC, gastric cancer; AG, atrophic gastritis; CON, control; Hp, *H. pylori*; B-D test, Breslow-Day test.

The association between each SNP and risk of AG and GC was estimated by calculating ORs and their 95% CIs by multivariate logistic regression with adjustments for gender, age, and *H. pylori* infection status.

Abbreviations: GC, gastric cancer; AG, atrophic gastritis; CON, control; Hp, *H. pylori*; B-D test, Breslow-Day test.

### Haplotype analysis

Haplotypes and their frequencies for each gene were inferred by SHEsis online software based on observed genotypes (Table 3). *ERCC5* CGTAG, *XPA* CTC, and *DDB2* GTAC and GTAG haplotypes were observed to alter the AG risk, while *ERCC5* CGCTG, TATAG, and TGTAG, *XPA* TCC, *XPC* GCAAG, and *DDB2* GTAC and GTAG haplotypes conferred an altered risk to GC. After Bonferroni correction for multiple testing, the *DDB2* GTAG haplotype remained significantly associated with disease risk at each step of the CON→AG→GC development (AG vs CON: OR=2.88, *P* =7.51 × 10^−7^; GC vs AG: OR=2.90, *P* =5.68 × 10^−15^; GC vs CON: OR=8.42, *P* =2.22 × 10^−15^); the *DDB2* GTAC haplotype was associated with a reduced risk of GC compared with AG (OR=0.72, *P* =3.65 × 10^−6^), and a reduced risk of GC compared with CON (OR=0.63, *P* =8.31 × 10^−12^); while the *XPC* GCAAG haplotype conferred an increased risk of GC compared with AG (OR=1.88, *P* =6.98 × 10^−4^).

**Table 3:** Results of haplotype analysis

		Gastric mucosa status			CON→AG		AG→GC		CON→GC	
Gene	Haplotype	GC(%)	AG(%)	CON(%)	OR(95%CI)	P	OR(95%CI)	P	OR(95%CI)	P
*ERCC1*	CAAAGC	692.27(39.0)	688.12(41.1)	711.71(38.6)	1.11(0.97-1.28)	0.131	0.92(0.80-1.05)	0.207	1.01(0.88-1.16)	0.900
	CCAAGC	54.02(3.0)	43.19(2.6)	40.47(2.2)	/	/	1.19(0.79-1.78)	0.408	1.39(0.92-2.10)	0.115
	CCAATA	57.27(3.2)	47.53(2.8)	67.92(3.7)	0.76(0.52-1.11)	0.157	1.14(0.77-1.69)	0.505	0.87(0.61-1.24)	0.434
	CCGCTA	485.47(27.3)	471.43(28.2)	524.91(28.5)	0.98(0.85-1.14)	0.806	0.96(0.83-1.12)	0.603	0.94(0.81-1.09)	0.392
	TCACGA	394.30(22.2)	338.82(20.2)	394.59(21.4)	0.93(0.79-1.09)	0.374	1.13(0.96-1.33)	0.149	1.04(0.89-1.22)	0.607
*ERCC2*	CTGC	96.50(5.4)	95.68(5.8)	78.99(4.3)	1.34(0.99-1.82)	0.060	0.95(0.71-1.27)	0.708	1.27(0.94-1.72)	0.128
	CTGG	750.54(42.3)	700.52(42.1)	816.64(44.9)	0.89(0.77-1.01)	0.076	1.01(0.89-1.16)	0.838	0.90(0.79-1.03)	0.111
	CTTC	710.61(40.1)	664.07(40.0)	724.72(39.8)	1.00(0.87-1.14)	0.962	1.01(0.88-1.16)	0.869	1.01(0.88-1.15)	0.904
	CTTG	74.34(4.2)	78.71(4.7)	66.65(3.7)	1.30(0.93-1.82)	0.121	0.88(0.64-1.22)	0.455	1.15(0.82-1.61)	0.418
	TGGG	95.46(5.4)	86.44(5.2)	83.44(4.6)	1.14(0.83-1.55)	0.419	1.04(0.77-1.41)	0.790	1.18(0.88-1.60)	0.275
*ERCC3*	AAC	187.95(10.6)	194.00(11.6)	188.98(10.3)	1.14(0.92-1.41)	0.218	0.90(0.73-1.12)	0.353	1.03(0.83-1.28)	0.765
	AGT	532.84(30.0)	500.97(30.0)	572.98(31.2)	0.94(0.81-1.09)	0.409	1.01(0.87-1.16)	0.941	0.95(0.82-1.09)	0.446
	GGC	1018.86(57.3)	946.97(56.6)	1038.96(56.6)	1.00(0.87-1.14)	0.992	1.04(0.91-1.19)	0.599	1.04(0.91-1.19)	0.597
*ERCC4*	CCT	362.77(20.6)	357.90(21.5)	382.91(21.1)	1.03(0.87-1.21)	0.757	0.95(0.81-1.12)	0.535	0.97(0.83-1.15)	0.749
	TGC	380.99(21.6)	355.45(21.3)	398.32(21.9)	0.97(0.82-1.14)	0.685	1.02(0.87-1.20)	0.819	0.99(0.84-1.16)	0.859
	TGT	1007.01(57.2)	944.55(56.7)	1028.68(56.6)	1.01(0.88-1.15)	0.935	1.02(0.89-1.17)	0.748	1.03(0.90-1.17)	0.681
*ERCC5*	CGCAG	209.08(12.0)	205.77(12.5)	213.66(11.8)	1.06(0.86-1.30)	0.580	0.96(0.78-1.18)	0.721	1.02(0.83-1.25)	0.845
	CGCTG	162.74(9.3)	117.04(7.1)	139.44(7.7)	0.91(0.71-1.18)	0.471	**1.36(1.06-1.74)**	**0.015**	1.24(0.98-1.57)	0.078
	CGTAG	136.67(7.8)	112.95(6.8)	161.47(8.9)	**0.75(0.58-0.96)**	**0.022**	1.17(0.90-1.51)	0.246	0.87(0.69-1.10)	0.252
	TATAG	614.64(35.3)	650.29(39.4)	688.84(38.0)	1.05(0.92-1.21)	0.471	**0.84(0.73-0.97)**	**0.018**	0.89(0.77-1.02)	0.090
	TGTAA	84.11(4.8)	88.92(5.4)	108.21(6.0)	0.89(0.67-1.19)	0.436	0.90(0.66-1.22)	0.481	0.80(0.60-1.07)	0.132
	TGTAG	502.97(28.9)	453.82(27.5)	467.50(25.8)	1.08(0.93-1.26)	0.298	1.08(0.93-1.25)	0.323	**1.17(1.01-1.36)**	**0.039**
*XPA*	CTC	370.15(20.8)	391.46(23.3)	369.03(19.9)	**1.22(1.04-1.44)**	**0.014**	0.87(0.74-1.02)	0.086	1.06(0.90-1.25)	0.460
	TCC	903.16(50.9)	792.39(47.2)	919.38(49.5)	0.91(0.80-1.04)	0.157	**1.16(1.02-1.33)**	**0.026**	1.06(0.93-1.21)	0.401
	TTC	334.90(18.9)	327.15(19.5)	374.68(20.2)	0.96(0.81-1.13)	0.593	0.96(0.81-1.14)	0.663	0.92(0.78-1.09)	0.322
	TTT	157.88(8.9)	162.39(9.7)	185.24(10.0)	0.97(0.77-1.21)	0.752	0.91(0.73-1.15)	0.440	0.88(0.71-1.10)	0.267
*XPC*	CCAAC	477.24(27.0)	455.68(27.4)	504.16(27.4)	1.00(0.86-1.17)	0.967	0.98(0.84-1.14)	0.789	0.98(0.85-1.14)	0.816
	GCAAG	86.07(4.9)	44.15(2.7)	64.99(3.5)	0.75(0.51-1.10)	0.139	**1.88(1.30-2.72)**	**6.98 × 10^−4^**	**1.40(1.01-1.95)**	**0.044**
	GCCAC	647.59(36.6)	625.30(37.5)	659.35(35.8)	1.08(0.94-1.24)	0.254	0.96(0.83-1.10)	0.554	1.04(0.91-1.19)	0.582
	GTAAC	526.97(29.8)	509.63(30.6)	584.35(31.7)	0.95(0.83-1.10)	0.505	0.96(0.83-1.11)	0.592	0.92(0.79-1.06)	0.220
*DDB2*	ACGG	438.89(24.8)	420.58(25.2)	446.33(24.0)	1.06(0.91-1.24)	0.432	0.97(0.83-1.13)	0.691	1.04(0.90-1.22)	0.580
	ATAC	65.19(3.7)	42.61(2.5)	51.55(2.8)	/	/	1.45(0.98-2.15)	0.062	1.34(0.93-1.94)	0.120
	ATGG	66.77(3.8)	67.10(4.0)	70.91(3.8)	1.05(0.75-1.48)	0.763	0.93(0.66-1.31)	0.675	0.99(0.70-1.39)	0.947
	GCGG	39.01(2.2)	48.70(2.9)	57.12(3.1)	0.95(0.64-1.40)	0.779	/	/	0.71(0.47-1.07)	0.103
	GTAC	855.35(48.3)	929.72(55.6)	1108.04(59.6)	**0.84(0.73-0.96)**	**0.013**	**0.72(0.63-0.83)**	**3.65 × 10^−6^**	**0.63(0.55-0.72)**	**8.31 × 10^−12^**
	GTAG	206.44(11.7)	72.37(4.3)	28.73(1.5)	**2.88(1.86-4.47)**	**7.51 × 10^−7^**	**2.90(2.20-3.82)**	**5.68 × 10^−15^**	**8.42(5.67-12.51)**	**2.22 × 10^−15^**
	GTGG	86.99(4.9)	77.40(4.6)	84.57(4.5)	1.02(0.74-1.40)	0.912	1.06(0.77-1.44)	0.739	1.09(0.80-1.47)	0.604

For each comparison, genotypes other than the analyzed one were considered to be the reference, and all rare haplotypes with a frequency <0.03 were ignored.

Abbreviations: GC, gastric cancer; AG, atrophic gastritis; CON, control.

### NER pathway SNP-environment and SNP-SNP interactions

The interactive effects of NER gene polymorphisms and the environment were next explored. *XPA* rs2808668 and the drinking of alcohol were found to have an interactive effect on AG risk (P_interaction_=0.009), while *DDB2* rs326222, rs3781619, and rs830083 demonstrated interactive effects with smoking in the development of AG (P_interaction_=0.040, 0.005, and 0.007, respectively) (Table 4). *XPC* rs2607775 showed a significant interaction with smoking (P_interaction_=0.024) on GC risk: (CG+GG) genotype smokers had a 3.84-fold increased risk of developing GC (OR=3.84, 95%CI=1.71-8.64). No interaction was observed between SNPs and the drinking of alcohol nor between SNPs and *H. pylori* infection on GC risk.

**Table 4:** Interaction of NER pathway SNPs and environmental factors

	CON→AG				CON→GC			
Genotype	Nonsmoker	Smoker	Nondrinker	Drinker	Nonsmoker	Smoker	Nondrinker	Drinker
*XPA* rs2808668								
TT	1(ref)	0.94(0.58-1.55)	1(ref)	0.50(0.27-0.91)	1(ref)	1.63(0.96-2.75)	1(ref)	1.34(0.74-2.42)
CC+CT	0.94(0.68-1.30)	0.81(0.56-1.16)	0.81(0.60-1.09)	0.83(0.57-1.20)	1.10(0.76-1.60)	1.66(1.12-2.47)	1.01(0.70-1.44)	1.99(1.32-3.00)
	P_interaction_=0.995		**P_interaction_=0.009**		P_interaction_=0.900		P_interaction_=0.221	
*XPC* rs2607775								
CC	1(ref)	0.87(0.68-1.13)	1(ref)	0.91(0.68-1.20)	1(ref)	1.42(1.09-1.86)	1(ref)	1.78(1.33-2.38)
CG+GG	0.78(0.46-1.32)	0.77(0.27-2.18)	0.86(0.51-1.44)	0.47(0.15-1.52)	0.93(0.52-1.67)	3.84(1.71-8.64)	1.18(0.68-2.07)	2.71(1.17-6.28)
	P_interaction_=0.544		P_interaction_=0.447		**P_interaction_=0.024**		P_interaction_=0.650	
*DDB2* rs326222								
CT+TT	1(ref)	0.84(0.65-1.10)	1(ref)	0.91(0.68-1.21)	1(ref)	1.50(1.15-1.95)	1(ref)	1.85(1.39-2.48)
CC	1.16(0.70-1.95)	2.27(1.00-5.16)	1.58(0.96-2.60)	1.13(0.48-2.69)	0.79(0.41-1.51)	1.81(0.71-4.62)	1.11(0.59-2.07)	1.51(0.60-3.80)
	**P_interaction_=0.040**		P_interaction_=0.885		P_interaction_=0.551		P_interaction_=0.782	
*DDB2* rs3781619								
AG+AA	1(ref)	0.79(0.61-1.04)	1(ref)	0.87(0.64-1.17)	1(ref)	1.52(1.16-2.00)	1(ref)	1.87(1.39-2.51)
GG	0.93(0.62-1.41)	1.67(0.94-2.98)	1.16(0.79-1.71)	1.22(0.62-2.38)	0.76(0.46-1.25)	1.19(0.59-2.39)	0.89(0.55-1.45)	1.25(0.58-2.70)
	**P_interaction_=0.005**		P_interaction_=0.287		P_interaction_=0.674		P_interaction_=0.396	
*DDB2* rs830083								
CG+CC	1(ref)	0.80(0.61-1.05)	1(ref)	0.88(0.65-1.19)	1(ref)	1.71(1.29-2.29)	1(ref)	2.07(1.51-2.84)
GG	0.84(0.56-1.25)	1.31(0.76-2.25)	1.04(0.72-1.51)	0.95(0.51-1.76)	2.22(1.51-3.27)	2.72(1.58-4.67)	2.57(1.76-3.75)	3.11(1.75-5.50)
	**P_interaction_=0.007**		P_interaction_=0.372		P_interaction_=0.652		P_interaction_=0.167	

SNP-environment interaction effects were assessed from the likelihood ratio test, comparing the fit of the logistic model that included the main effects of sex, age, environment risk factor and genotype with a fully parameterized model containing the multiplicative interaction terms of genotype and environment risk factor.

Abbreviations: GC, gastric cancer; AG, atrophic gastritis; CON, control.

Supplementary Table S6 shows the effects of SNP-SNP interactions. No significant interaction was found between SNPs except for that between *XPA* rs2808668 and *DDB2* rs326222 in the development of GC from AG (P_interaction_=0.031).

## DISCUSSION

Identifying biomarkers associated with high GC risk has long been a research goal to help improve early disease detection. Although several previous studies have suggested that NER gene polymorphisms could alter GC susceptibility, to the best of our knowledge this is the first large-scale investigation of the relationship between SNPs of the entire NER gene pathway and risks of developing GC and AG. We found that the *DDB2* rs830083 GG genotype and *XPC* rs2607775 CG genotype were associated with increased GC risk, and haplotype analysis revealed that the *DDB2* GTAG haplotype significantly increased GC risk. Significant interactions between *XPA* rs2808668 and the drinking of alcohol, as well as between *DDB2* SNPs rs326222, rs3781619, and rs830083 and smoking, were identified in the pathogenesis of AG. Moreover, in GC development, *XPC* rs2607775 demonstrated a significant interaction with smoking. Thus, polymorphisms in *DDB2* and *XPC* are associated with increased risks of developing GC and AG.

*DDB2* encodes damage-specific DNA binding protein 2, which is responsible for damage recognition and the initiating NER. UV-induced DNA damage is recognized by a heterodimer formed from DDB1 and DDB2 [19]. Recent research has revealed that DDB2 is the downstream target of tumour suppressor genes p53 and *BRCA1* [20, 21], and it is known to play an important role in regulating p53 function and controlling p53-mediated apoptosis [22, 23]. Yoon et al. found that *DDB2*-defective mice have a high risk of developing spontaneous tumours, which is indicative of a protective role for DDB2 in cancer development [24].

Human *DDB2* is located on chromosome 11p12-p11, and the rs830083 C/G polymorphism is within its intronic region. Previously, rs830083 has been suggested to significantly increase the risk of lung cancer [25], but no study has yet investigated its association with GC. Here, carriers of the *DDB2* rs830083 GG genotype demonstrated significantly increased risks of developing AG (OR=1.48) and GC (OR=2.32) compared with the wild-type CC genotype, suggesting that this polymorphism affects different stages of gastric carcinogenesis. Considering the critical role of DDB2 in the NER pathway, this polymorphism could alter the function of DDB2, thereby affecting individual susceptibility to GC. However, the molecular mechanism has yet to be investigated by future studies. We further performed subgroup analysis and showed that the association of the GG genotype and GC was more significant in males, non-smokers, and non-drinkers, suggesting that the *DDB2* rs830083 polymorphism might have an improved predictive role in these subgroups. The consistent significant association seen in *H. pylori*-positive and -negative, as well as intestinal-type and diffuse-type GC indicated that the *DDB2* rs830083 polymorphism has a stable predictive effect despite *H. pylori* infection and GC type. However, as the samples for subgroup analysis were relatively small, further large-scale studies are required to confirm these preliminary findings.

XPC also plays an essential role in the damage recognition step of NER. In mammals, the heterotrimeric XPC complex composed of XPC, RAD23, and centrin-2 recognizes DNA distortions and is indispensable for initiating global genome NER [26]. *XPC*, located on chromosome 3p25, consists of 16 exons and 15 introns and encodes a DNA-binding protein of 940 amino acids that preferentially binds damaged DNA [27]. The *XPC* rs2607775 polymorphism is located upstream of the *XPC* regulatory region and its effect on transcription is largely unknown. In the present study, carriers of the *XPC* rs2607775 CG genotype had a 1.73-fold increased risk of GC than subjects with the wild-type CC genotype. Subgroup analysis found that the *XPC* rs2607775 CG genotype still demonstrated a significantly increased GC risk in males and *H. pylori*-negative subgroups. Moreover, individuals with both *DDB2* rs830083 and *XPC* rs2607775 risk genotypes had a 3.05-fold increased risk of GC (Fig.1), indicating that these two NER genes might serve as future joint predictive biomarkers for GC risk.

Previous studies have identified relationships between some NER gene SNPs and risk of GC in different populations. For example, the *ERCC1* rs2298881 and rs11615 polymorphisms were associated with a higher risk of GC in eastern Chinese individuals [28], but we only found the rs11615 polymorphism to be associated with an increased GC risk in *H. pylori*-positive northern Chinese subjects (Table S4). This might reflect the variation of *H. pylori* strains from different regions leading to differences in individual environmental exposure. Similarly, *ERCC2* rs13181 was reported to alter individual susceptibility to GC [29], but we observed no significant association either in the total or subgroup analysis of our study. Previous studies have drawn inconsistent conclusions regarding the association between *ERCC5* rs2296147 and GC risk [30, 31]. In the present study, we found *ERCC5* rs2296147 to be associated with increased GC risk in *H. pylori*-positive and diffuse-type GC subgroups (Table S4 and Table S5). The *ERCC5* rs2094258 AA genotype has been reported to be associated with decreased GC risk [31], which is consistent with our findings in *H. pylori*-negative and diffuse-type GC subgroups. Although *ERCC5* rs751402 and rs873601 have been linked with altered GC risk [28, 30], we did not investigate these two polymorphisms further because they were not in accordance with HWE. It is worth noting that XPA rs2808668 showed a protective effect in “CON to AG”, but a risk effect in “AG to GC”. We then performed stratified analysis and found that the risk effect in “AG to GC” was only found in diffuse-type GC but not in intestinal-type GC (Supplementary Table S5). As described by Correa's cascade, it is accepted that intestinal-type GC progresses stepwise from normal stomach, atrophic gastritis to carcinoma, while the pathogenesis of diffuse-type GC is still unclear[2]. Therefore, the findings of this study might indicate possible involvement of this polymorphism in the complex process of diffuse-type GC, and the underlying mechanisms still require further studies to elucidate.

Haplotype analysis covering SNPs of different regions would be effective at revealing the role of key NER factors and providing a pathway overview, but most previous studies only focused on a few SNPs rather than performing a combined analysis. In this study, we investigated the joint effect of multiple polymorphisms to determine the most important NER pathway factor associated with GC risk. The *DDB2* rs2029298-rs326222-rs3781619-rs830083 GTAG haplotype was significantly associated with disease risk at each step of CON→AG→GC development, while the *DDB2* GTAC haplotype was associated with a reduced risk of GC compared with CON. Moreover, the *XPC* rs1870134-rs2228000-rs2228001-rs2470352-rs2607775 GCAAG haplotype conferred an increased risk of GC compared with AG. These findings not only strongly suggest that *DDB2* and *XPC* are GC susceptibility genes, but also highlight the potential of predicting GC risk by detecting key NER pathway haplotypes. Considering that both DDB2 and XPC are critical for the damage recognition stage of NER, we speculate that this might be the crucial step that determines whether GC develops.

Apart from genetic factors, gastric carcinogenesis is also influenced by environmental factors such as *H. pylori* infection, smoking, and the consumption of alcohol, of which *H. pylori* is the best known environmental pathogenic factor [32]. Chronic *H. pylori* infection, smoking, and alcohol consumption can induce persistent inflammation and/or the generation of reactive oxygen species, which leads to DNA damage [33]. Thus, polymorphisms of key NER genes responsible for damage repair may have synergistic effects with *H. pylori* infection in the development of GC. Previous research found that *ERCC5* rs2296147 and rs2094258 interacted with *H. pylori* infection in the development of GC [31], and that *XPC* PAT(+/−) showed an interactive effect with smoking in GC [34]. However, no clear overview of the gene-environment interaction in the NER pathway has been provided. Our results suggested that *XPA* rs2808668 significantly interacted with alcohol consumption in the development of AG. Similarly, *DDB2* rs326222, rs3781619, and rs830083 SNPs showed significant interactions with smoking in AG development. *XPC* rs2607775 had an interactive effect with smoking in GC development, with (CG+GG) genotype smokers having a 3.84-fold increased risk of GC. NER is a complex, multi-step process, and different NER gene polymorphisms might function jointly in gastric carcinogenesis. However, no significant interaction was found between SNPs, except for an interaction between *XPA* rs2808668 and *DDB2* rs326222, in the development of GC from AG.

It is notable that polymorphisms demonstrating significant interactions with environmental factors are all located within genes responsible for the DNA damage recognition step of NER (*XPA*, *XPC*, and *DDB2*). This suggests that NER gene polymorphisms exert interactive effects with environmental factors during DNA damage recognition, thereby influencing an individual's capacity for DNA repair and susceptibility to GC. This finding may not only provide direction for further investigations into the molecular interactions between NER gene polymorphisms, but may also suggest a potential target for the primary prevention of GC in the future. This could take the form of lifestyle interventions for carriers of high risk GC genotypes and the control of environmental factors. Thus, avoidance of a high salt diet, cessation of smoking and drinking alcohol, and eradication of *H. pylori* infection, which may all interact with a susceptible genotype to increase GC risk, could be an effective method for disease prevention. One limitation for this study is that the functions and mechanisms of the associated SNPs of this pathway were not analyzed, which need further functional studies to elucidate. Secondly, the gender and age of subjects between cases and controls were not matched although these factors have been adjusted in the multivariable analysis, which might influence the results of the disease association. In addition, because the information of smoking and drinking status was not available for all of the included subjects, we did not adjust the smoking and drinking status in multivariate logistic regression.

In summary, we found for the first time that the two polymorphisms *DDB2* rs830083 and *XPC* rs2607775, affecting the damage recognition step of the NER pathway, were significantly associated with increased GC risk, and that the combined detection of these two polymorphisms demonstrated an even higher GC risk (OR=3.05). Future systematic SNP screening and GWAS studies focusing on the relevant NER pathway may identify more susceptible genes and their interactions with environmental factors. It is anticipated that this would not only provide an effective basis for the early warning of high GC risk individuals with the aim of individualized prevention, but also reveal important molecular mechanisms and novel therapies for GC.

## MATERIALS AND METHODS

### Study population

A total of 2686 subjects were selected for inclusion in the present study. All enrolled individuals were unrelated Han ethnic Chinese living in northern China, who were recruited from a health check program for GC screening or hospitals in Zhuanghe and Shenyang of Liaoning Province, China between 2002 and 2013. Patients with a history of other malignancies were excluded. The gastroscopy examination was performed by experienced endoscopists. Four biopsy specimens were obtained from the body, angulus, antrum, and site of the lesion. Each subject was assigned a global diagnosis based on the most severe lesion among the four biopsy specimens, and this was confirmed independently by two gastrointestinal pathologists. Histopathological findings were assessed according to the Consensus on Chronic Gastritis formulated at the National Symposium in combination with the updated Sydney System [15] and the World Health Organization criteria.

Three groups of subjects were retrospectively selected from the total participants based on their baseline diagnosis: (i) a control (CON) group of individuals with normal stomach and subjects with slight or moderate gastritis without atrophic or intestinal metaplasia lesions (n=937); (ii) an atrophic gastritis (AG) group with or without intestinal metaplasia (n=851); and (iii) a gastric cancer (GC) group (n=898). Subjects were excluded from the CON group if they had gastric erosion, peptic ulcer disease, gastric polyps, adenomas, or diseases related to or causing predisposition to cancer.

Each participant was interviewed face-to-face by trained interviewers using a standardized questionnaire. Written informed consent was obtained from each participant. Data including gender, age, history of illness, native origin, pathological diagnosis, and status of smoking, alcohol consumption, and *H. pylori* infection were coded in a specific databank. Individuals who smoked at least once a day for more than 1 year were defined as smokers, and the others were defined as non-smokers. Subjects who consumed alcohol at least once a week for more than 1 year were defined as drinkers, while the remainder were non-drinkers.

Fasting blood samples (5 ml) were collected from each patient for DNA extraction and measuring serum *H. pylori* immunoglobulin G levels. This study was approved by the human ethics review committee of China Medical University (Shenyang, China).

### Candidate genes and SNP selection

We extracted genotype data from extended NER gene regions encompassing 5 kb of upstream and downstream flanking sequences from the HapMap Chinese Han Beijing population (Release 27, Phase I + II + III, http://www.HapMap.org). Haploview software (http://www.broadinstitute.org/mpg/haploview) was used to minimize the number of SNPs required to be genotyped, providing an important shortcut to carry out candidate gene association studies in a particular population. Tag SNPs were chosen based on pairwise linkage disequilibrium information to maximally capture (r^2^ > 0.8) common or rare variants (minor allele frequency [MAF] > 0.05) by Haploview 4.2. FastSNP Search was used to predict the potential SNP function (leading to amino acid substitutions, altering splicing or transcription factor-binding motifs, acting as intronic enhancers) [16, 17]. A total of 39 SNPs covering eight key NER pathway genes (*ERCC1*, *ERCC2*, *ERCC3*, *ERCC4*, *ERCC5*, *XPA*, *XPC*, and *DDB2*) were selected by integrating these two publicly available tools. The flow chart of the detailed SNP selecting strategy was summarized in Supplementary Figure 1.

### Genotyping assay

Genomic DNA was isolated from blood samples using routine phenol-chloroform extraction and then diluted to working concentrations (50 ng/ μ l) for genotyping. Samples were placed randomly on the 384-well plates and blinded for disease status. The design of the assay and SNP genotyping were performed by Bio Miao Biological Technology (Beijing, China) using the Sequenom MassARRAY platform (Sequenom, San Diego, CA) according to the manufacturer's instructions. The average genotyping rate was 99.3% and the results of all duplicated samples were 100% consistent.

### H. pylori serology

*H. pylori* serology was performed to examine the status of *H. pylori* infection using an enzyme-linked immunosorbent assay. *H. pylori* immunoglobulin G concentrations of serum samples were detected by an ELISA kit (Biohit, Helsinki, Finland) according to the manufacturer's instructions. A numerical reading exceeding 34 enzyme immune-units was considered to be *H. pylori*-positive.

### Statistical analysis

HWE for each SNP was first evaluated among control subjects using either the chi-square (χ^2^) test or Fisher's exact test. We excluded SNPs that deviated from HWE from subsequent association analysis. Age differences between groups were assessed using the analysis of variance test. The χ^2^ test was applied to evaluate differences in categorical variables including gender, *H. pylori* infection, smoking status, and consumption of alcohol. The association between each SNP and risk of AG and GC was estimated by calculating ORs and their 95% CIs by multivariate logistic regression with adjustments for gender, age, and *H. pylori* infection status. Recessive model, dominant model, co-dominant model and additive model were adopted. Stratified analysis by gender, Lauren's classification, *H. pylori* infection, and smoking and alcohol consumption status was also conducted. To compare the SNP effect on the risks of AG and GC between different subgroups, the Breslow-Day test was used to assess the homogeneity of stratum-specific ORs across different subgroups. Statistical significance was set as *P* ≤ 0.10 for this test. To limit spurious findings, we used the Bonferroni correction for multiple comparisons considering significance thresholds for SNP association as *P* =1.5 × 10^−3^ (0.05/33 SNPs) and for haplotype association as P=6.25 × 10^−3^ (0.05/8 genes). This is a fairly stringent correction given that not all of the SNPs analyzed may be independent of each other because of linkage disequilibrium. SNP-environment interaction effects were assessed from the likelihood ratio test, comparing the fit of the logistic model that included the main effects of sex, age, environment risk factor and genotype with a fully parameterized model containing the multiplicative interaction terms of genotype and environment risk factor. Likelihood ratio test was also performed to assess the SNP-SNP interaction effects on the risk of GC by comparing the model only involving main effects of gender, age, genotype with the full model which also contained the SNP-SNP interaction term. The abovementioned statistical analyses for SNP association were performed using SPSS 16.0 software (SPSS, Chicago, IL). Haplotype association analyses were performed for all investigated genes by SHEsis online software (http://analysis.bio-x.cn/myAnalysis.php) [18] to explore the relation between haplotype and disease risk. For each comparison, genotypes other than the analyzed one were considered to be the reference, and all rare haplotypes with a frequency < 0.03 were ignored.

## SUPPLEMENTARY FIGURES AND TABLES










