# Supplementary material for: Association of nucleotide excision repair pathway gene polymorphisms with gastric cancer and atrophic gastritis risks
Source: Oncotarget. 2016 Jan 9;7(6):6972–83. doi: 10.18632/oncotarget.6853 (PMC4872762; doi:10.18632/oncotarget.6853)
Supplement: Supplementary file 1 [file oncotarget-07-6972-s001.pdf]

SUPPLEMENTARY FIGURE AND TABLES

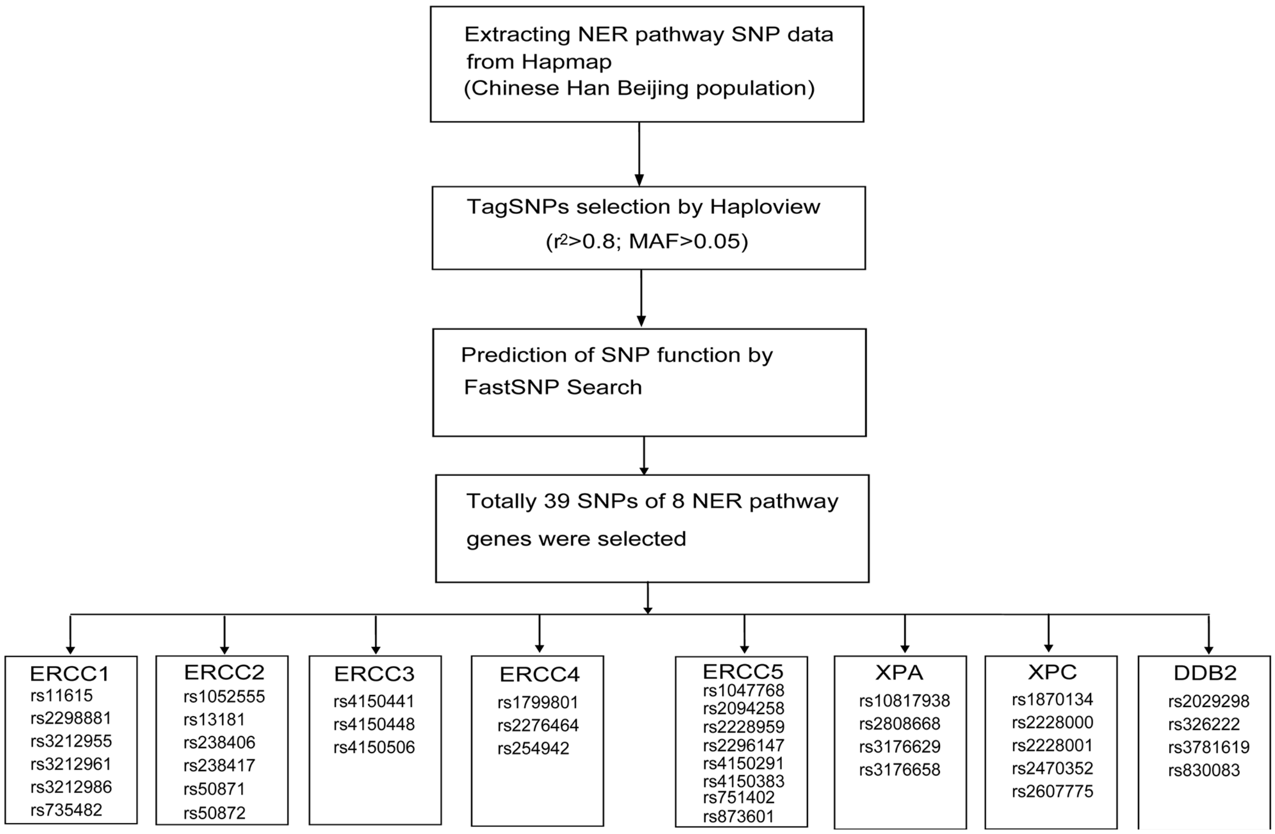

Supplementary Figure S1: Flow chart of the detailed SNP selecting strategy.

Supplementary Table S1: Details of 39 genotyped SNPs in NER pathway

Gene	dbSNP number	Base change	SNP location	MAF				P for HWE	Genotyping rate(%)
				In database	GC	AG	CON		
ERCC1	rs11615	C > T	Exon	0.243	0.238	0.218	0.238	0.984	99.6
	rs2298881	C > A	Promoter	0.444	0.400	0.424	0.397	0.415	99.4
	rs3212955	A > G	Intron	0.289	0.300	0.304	0.301	0.995	99.6
	rs3212961	C > A	Intron	0.453	0.472	0.486	0.471	0.945	99.6
	rs3212986	G > T	3′ Untranslated region	0.310	0.318	0.322	0.335	0.379	99.6
	rs735482	A > C	3′ Untranslated region	0.427	0.442	0.457	0.433	0.479	99.3
ERCC2	rs1052555	C > T	Exon	0.104	0.066	0.060	0.058	0.502	99.7
	rs13181	T > G	Exon	0.095	0.080	0.073	0.073	0.347	99.7
	rs238406	G > T	Exon	0.407	0.454	0.456	0.446	0.790	99.3
	rs238417	G > C	Intron	0.488	0.457	0.457	0.444	0.372	98.3
	rs50871	T > G	Intron	0.279	0.368	0.336	0.329	**< 0.001**	99.6
	rs50872	C > T	Intron	0.190	0.217	0.197	0.199	**0.040**	99.6
ERCC3	rs4150441	G > A	Intron	0.444	0.421	0.433	0.434	0.823	99.2
	rs4150448	G > A	Intron	0.109	0.106	0.115	0.106	0.582	99.4
	rs4150506	C > T	Intron	0.320	0.305	0.302	0.314	0.565	99.1
ERCC4	rs1799801	T > C	Exon	0.237	0.211	0.218	0.220	0.262	99.6
	rs2276464	G > C	3′ Untranslated region	0.275	0.207	0.215	0.212	0.532	99.3
	rs254942	T > C	Intron	0.241	0.220	0.214	0.221	0.282	98.2
ERCC5	rs1047768	T > C	Exon	0.241	0.301	0.279	0.291	0.062	99.5
	rs2094258	G > A	Promoter	0.383	0.363	0.395	0.389	0.563	98.1
	rs2228959	C > A	Exon	0.062	0.044	0.049	0.050	**0.011**	99.6
	rs2296147	T > C	5′ Upstream	0.201	0.218	0.205	0.207	0.295	99.1
	rs4150291	A > T	Intron	0.081	0.094	0.075	0.078	0.123	99.6
	rs4150383	G > A	Intron	0.088	0.052	0.054	0.061	0.780	99.2
	rs751402	C > T	Promoter	0.367	0.344	0.325	0.318	**0.017**	96.8
	rs873601	G > A	3′ Untranslated region	0.496	0.490	0.480	0.475	**0.016**	99.2
XPA	rs10817938	T > C	5′ Upstream	0.208	0.214	0.235	0.201	0.955	99.4
	rs2808668	T > C	Intron	0.478	0.487	0.474	0.499	0.251	99.1
	rs3176629	C > T	Promoter	0.088	0.090	0.100	0.102	0.660	99.6

(*Continued*)

Gene	dbSNP number	Base change	SNP location	MAF In database	MAF GC	MAF AG	MAF CON	P for HWE	Genotyping rate(%)
	rs3176658	C > T	Intron	0.256	/	/	/	/	98.9
XPC	rs1870134	G > C	5′ Upstream	0.244	0.272	0.277	0.276	0.176	99.4
	rs2228000	C > T	Exon	0.325	0.305	0.310	0.323	0.828	99.0
	rs2228001	A > C	Exon	0.372	0.365	0.376	0.360	0.898	99.4
	rs2470352	A > T	3′ UTR	0.058	0.004	0.002	0.003	0.922	99.5
	rs2607775	C > G	5′ Upstream	0.089	0.054	0.030	0.039	0.733	99.7
DDB2	rs2029298	G > A	Promoter	0.354	0.327	0.321	0.310	0.412	99.4
	rs326222	T > C	Intron	0.274	0.272	0.285	0.276	0.102	99.5
	rs3781619	A > G	Intron	0.383	0.359	0.370	0.357	0.483	99.5
	rs830083	C > G	Intron	0.367	0.480	0.414	0.373	0.330	99.4

MAF for Chinese in Hapmap daabase(www. hapmap.org)
Abbreviations: GC, gastric cancer; AG, atrophic gastritis; CON, control; MAF, minor allele frequency; HWE, Hardy-Weinberg equilibrium.

Supplementary Table S2: Baseline characteristics of the study population

Variables	GC(%)	AG(%)	CON(%)	P
Total	898	851	937	
Age(mean ± SD, year)	59.41 ± 11.39	55.94 ± 9.92	53.70 ± 9.97	< 0.001
Range	21–87	16–84	17–85	
Gender				< 0.001
Male	639(71.2)	478(56.2)	510(54.4)	
Female	259(28.8)	373(43.8)	427(45.6)	
Lauren's classification				
Intestinal-type GC	269(37.3)	/	/	
Diffuse-type GC	453(62.7)	/	/	
H. pylori infection				< 0.001
Positive	454(50.6)	502(59.0)	274(29.2)	
Negative	444(49.4)	349(41.0)	663(70.8)	
Smoking				< 0.001
Smoker	177(43.5)	160(31.0)	212(33.5)	
Nonsmoker	230(56.5)	356(69.0)	420(66.5)	
Drinking				< 0.001
Drinker	134(36.5)	114(22.2)	153(24.2)	
Nondrinker	233(63.5)	400(77.8)	478(75.8)	

Abbreviations: GC, gastric cancer; AG, atrophic gastritis; CON, control.

Supplementary Table S3: Results of the association between NER pathway polymorphisms and risks of AG and GC
See Supplementary File 1

Supplementary Table S4: Subgroup analysis based on *H. pylori* infection
See Supplementary File 2

Supplementary Table S5: Subgroup analysis based on intestinal and diffuse GC
See Supplementary File 3

Supplementary Table S6: Results of SNP-SNP interactions
See Supplementary File 4
